# Foot dorsum thermal quantitative sensory testing thresholds in healthy Vietnamese adults: Reference data

**DOI:** 10.1016/j.cnp.2026.06.009

**Published:** 2026-06-25

**Authors:** Phan Hoang Phuong Khanh, Pham Dinh San, Phan Hoang Dang Khoa, Nguyen Huu Cong, Nguyen Le Viet Hung, Nguyen Le Trung Hieu

**Affiliations:** a Department of Neurology, School of Medicine*,* University of Medicine and Pharmacy at Ho Chi Minh City*,* Ho Chi Minh City*,* Viet Nam; b Department of Neurology, International Neurosurgery Hospital*,* Ho Chi Minh City*,* Viet Nam; c Military Hospital 175*,* Viet Nam; d Hanh Phuc International Hospital*,* Hoan My Medical Group*,* Ho Chi Minh City*,* Viet Nam; e University of Medicine and Pharmacy at Ho Chi Minh City, Ho Chi Minh City, Viet Nam

**Keywords:** Quantitative sensory testing, Small fiber neuropathy, Reference data, Vietnamese population, Metabolic factors, Method of limits

## Abstract

**Objective:** To provide preliminary reference data for thermal quantitative sensory testing (QST) at the foot dorsum in healthy Vietnamese adults and to examine associations with demographic and metabolic variables.

**Methods:** This cross-sectional study included 68 healthy Vietnamese adults (23–80 years). Participants were screened to exclude diabetes and prediabetes according to ADA 2024 criteria. Cold Detection Threshold (CDT), Warm Detection Threshold (WDT), and Heat Pain Threshold (HPT) were assessed bilaterally at the foot dorsum using the Q-Sense device and the Method of Limits protocol. Reference intervals (2.5th–97.5th percentiles) were estimated using non-parametric methods. Multivariable linear regression was used to evaluate associations with age, sex, BMI, LDL-C, triglycerides, and HbA1c.

**Results:** Bilateral symmetry supported the use of averaged bilateral values. Age was independently associated with all QST parameters (CDT β = −0.0299; WDT β = 0.0801; HPT β = 0.0739; all *p* < 0.001). In the reduced model, BMI was associated with CDT (β = 0.0929, *p* = 0.030), but this was not observed in the full model. LDL-C was associated with WDT in both models (β = 0.0122, *p* = 0.007; β = 0.0121, *p* = 0.011). Sex was not consistently associated with any parameter.

**Conclusions:** This study provides preliminary reference data for thermal QST in a Vietnamese population. Age was the most determinant of thermal thresholds. LDL-C was associated with warm detection thresholds, while BMI was not significant in the full model.

**Significance:** These data may support interpretation of thermal QST in clinical practice.

## Highlights

• Preliminary reference data for foot dorsum thermal thresholds in Vietnamese adults.
• Age was the strongest determinant of foot dorsum thermal thresholds.
• Low-density lipoprotein cholesterol was associated with warm detection thresholds.

## 1 Introduction

Polyneuropathy may involve small, large, or mixed fiber dysfunction depending on the underlying etiology, and small fiber involvement is commonly associated with sensory symptoms such as pain and thermal impairment (Taub and Woolf, 2024). However, conventional nerve conduction studies assess only the function of large myelinated fibers, thereby often failing to detect early-stage damage in small fiber pathology (Chong and Cros, 2004). Quantitative Sensory Testing has established itself as a psychophysical method for the non-invasive quantification of small fiber function (Backonja et al., 2013). Specifically, thermal QST evaluates small fiber function, including thinly myelinated Aδ fibers mediating cold detection and contributing to cold pain, and unmyelinated C fibers mediating warm detection and heat pain (Backonja et al., 2013; Chong and Cros, 2004).

Despite its clinical utility, the interpretation of QST results relies on appropriate reference values, which are significantly influenced by methodological and demographic variables (Rolke et al., 2006). The “Method of Limits,” which inherently incorporates reaction time, is frequently preferred in clinical settings due to its time efficiency and ease of administration (Boland-Freitas et al., 2016; Heldestad Lilliesköld and Nordh, 2018). Using this method, age is consistently identified as the primary determinant of thermal sensory thresholds, with sensory acuity declining progressively over the lifespan in a length-dependent manner, particularly at distal sites such as the foot (Heldestad Lilliesköld and Nordh, 2018; Lin et al., 2005; Zhi et al., 2024). The influence of sex remains heavily debated; while some studies have demonstrated superior thermal sensitivity in females (Cosentino et al., 2023; Harju, 2002), large multicenter trials suggest that the robust effect of aging may overshadow subtle sex-related differences at distal extremities (Rolke et al., 2006).

Crucially, ethnic and geographical disparities in somatosensory profiles have been well documented, revealing that reference values derived from Caucasian or Hispanic populations may not be directly applicable to Asian cohorts without risking diagnostic misclassification (González-Duarte et al., 2016; Pan et al., 2024). Furthermore, establishing a well-characterized reference population requires careful assessment of metabolic status, particularly glycemic regulation (American Diabetes Association Professional Practice Committee, 2024; Taub and Woolf, 2024). To date, standardized normative QST data for the Vietnamese population are completely lacking. To address this critical gap, this study aimed to provide preliminary reference data for thermal perception thresholds (Cold Detection, Warm Detection, and Heat Pain) at the foot dorsum in healthy Vietnamese adults using the Q-Sense device via the Method of Limits. Additionally, we explored associations between demographic and metabolic variables and thermal thresholds, providing preliminary population-specific reference data for clinical interpretation.

## 2 Materials and methods

### 2.1 Participants and procedure

A cross-sectional study was conducted on 68 healthy Vietnamese volunteers (34 males and 34 females; mean age 51.9 ± 13.7 years, range 23–80 years). Participants were recruited via convenience sampling from the local community, hospital staff, and relatives of inpatients at the International Neurosurgery Hospital (Ho Chi Minh City, Vietnam). Inclusion criteria required normal fasting venous glucose and HbA1c levels according to the American Diabetes Association (ADA) diagnostic criteria (American Diabetes Association Professional Practice Committee, 2024). Participants with diabetes or prediabetes, as well as those with frank obesity (body mass index (BMI) ≥30 kg/m^2^), were excluded, while dyslipidemia was not used as an exclusion criterion. We excluded individuals with peripheral neuropathy, chronic pain syndromes, alcohol abuse, cognitive impairment, or the use of medications affecting the central or peripheral nervous system within the preceding 72 h.

### 2.2 Thermal testing

Thermal QST was performed using the Q-Sense device (Medoc Ltd., Ramat Yishai, Israel) equipped with a standard 30 × 30 mm Peltier thermode. All examinations were conducted in a quiet, temperature-controlled room (22–24 °C). Participants were allowed to acclimatize to the environment for at least 15 min before testing. The assessments were conducted using the reaction-time inclusive “Method of Limits” (MLI), which is commonly used in clinical settings due to its efficiency (Heldestad Lilliesköld and Nordh, 2018). This method includes reaction time and may influence threshold estimation, particularly in older individuals. To minimize this effect, standardized testing procedures and repeated measurements were used. The baseline adaptation temperature was set to 32 °C. The rate of temperature change was 1 °C/s for all stimuli, with safety cut-off limits were set at 16 °C and 50 °C to prevent tissue damage. These parameters were selected in accordance with established QST protocols to ensure standardization and comparability across studies (Backonja et al., 2013; Rolke et al., 2006). The testing sequence was fixed: Warm Detection Threshold (WDT), Cold Detection Threshold (CDT), and Heat Pain Threshold (HPT). Cold Pain Threshold (CPT) was initially attempted using the lowest available temperature of the device (12 °C); however, some participants did not report pain at this level, suggesting a floor effect and unreliable estimation. Therefore, CPT was not included in the final analysis. The exclusion of CPT may limit the completeness of small fiber assessment. Participants were instructed to press an electrical switch immediately upon perceiving a change in temperature (for WDT and CDT) or at the first sensation of pain (for HPT). Each threshold was determined as the arithmetic mean of three to four consecutive stimuli.

### 2.3 Testing sites

To evaluate the length-dependent function of small nerve fibers, measurements were performed bilaterally at the distal lower extremities. The thermode was firmly attached to the dorsum of the foot, specifically proximal to the 2nd and 3rd metatarsophalangeal joints. Bilateral assessments were conducted to assess sensory symmetry in this healthy cohort, thereby providing a baseline for relative side-to-side diagnostic comparisons in future clinical practice (Kemler et al., 2000).

### 2.4 Data conditioning and statistical analyses

Data conditioning and statistical analyses were performed using R statistical software. The distributional characteristics of QST parameters were initially evaluated using histograms, kernel density plots, and the Shapiro-Wilk test for normality. Bilateral symmetry was assessed using the paired *t*-test for normally distributed variables (WDT) and the Wilcoxon signed-rank test for variables with mild deviations from normality (CDT, HPT). Given the high degree of inter-side symmetry, data from the left and right feet were averaged for subsequent analyses.

Descriptive statistics for central tendency were reported as mean ± standard deviation (SD) or median with interquartile range (IQR), as appropriate. Descriptive reference ranges were estimated using non-parametric methods (2.5th–97.5th percentiles) (Rolke et al., 2006). Confidence intervals for the percentile limits were obtained using bootstrap resampling (5000 iterations). Given the sample size, particularly within age strata, these estimates are preliminary and should be interpreted with caution.

To evaluate associations between demographic and metabolic variables and QST parameters, univariable analyses were first performed using Spearman's rank correlation (ρ). Multivariable linear regression analyses were subsequently conducted. A full model (Model 1) was constructed including age, sex, height, BMI, low-density lipoprotein cholesterol (LDL-C), triglycerides, and HbA1c as predictors. Model assumptions, including linearity and multicollinearity, were assessed. Multicollinearity was evaluated using variance inflation factors (VIF), with all predictors showing low VIF values (<2), indicating no significant multicollinearity. To simplify the model, a reduced model (Model 2) was constructed by retaining age and sex a priori, along with variables identified as statistically or clinically relevant in Model 1.

### 2.5 Ethics statement

The study protocol was conducted in strict accordance with the ethical principles outlined in the Declaration of Helsinki. All study procedures were reviewed and formally approved by the Biomedical Research Ethics Committee of the University of Medicine and Pharmacy at Ho Chi Minh City (Approval No. 293/HDDD-DHYD, dated 01/02/2024). Written informed consent was obtained from all participants prior to their enrollment and before any clinical assessments or data collection were performed.

## 3 Results

### 3.1 Demographic and clinical characteristics

The study included 68 participants with a mean age of 51.9 ± 13.7 years (range: 23–80 years). Age groups were well distributed, and the sex ratio was balanced with equal numbers of males and females. Participants had a mean BMI of 22.7 ± 2.5 kg/m^2^ and demonstrated normal glycemic profiles, with mean HbA1c and postprandial glucose levels within reference ranges. Lipid parameters showed moderate variability, particularly for triglycerides. Detailed baseline characteristics are presented in Table 1.

**Table 1:** Baseline characteristics of study population (*n* = 68).

**Characteristic**	*Mean ± SD (Min - Max); n (%)*
**Age (years)**	51.91 ± 13.67 (23.00–80.00)
**Age group, n (%)**	
18–39	17 (25%)
40–59	32 (47%)
≥60	19 (28%)
**Sex, n (%)**	
Male	34 (50%)
Female	34 (50%)
**Height (m)**	1.58 ± 0.07 (1.47–1.72)
**Body mass index (kg/m**^**2**^**)**	22.72 ± 2.46 (17.90–27.34)
**HbA1c (%)**	5.37 ± 0.25 (4.50–5.70)
**Postprandial glucose (mg/dL)**	96.79 ± 9.24 (68.70–114.30)
**LDL-C (mg/dL)**	124.88 ± 36.93 (61.00–247.00)
**Triglycerides (mg/dL)**	170.62 ± 81.20 (56.00–449.00)

### 3.2 Distribution and bilateral symmetry of thermal sensory thresholds

The distributional properties of thermal quantitative sensory testing parameters are illustrated in Supplementary Fig. 1. Histograms with overlaid density curves and formal normality testing demonstrate that warm detection thresholds approximate a normal distribution, whereas cold detection and heat pain thresholds show mild deviations from normality. Detailed results of the normality assessment are provided in the Supplementary Appendix.

**Fig. 1:**
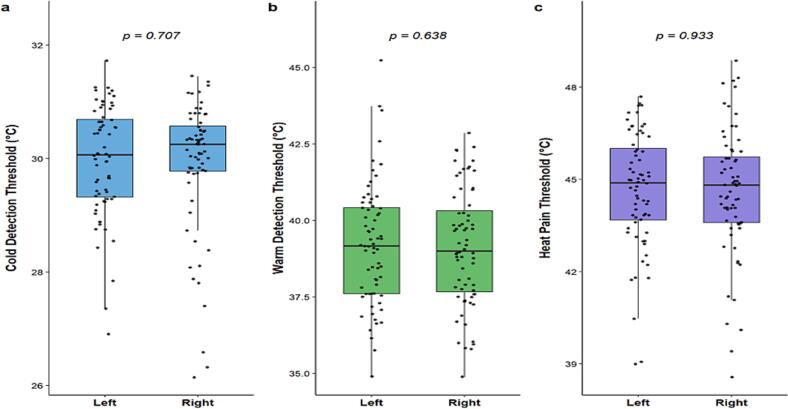
Bilateral comparison of thermal QST parameters at the foot dorsum. **Note. (**a) CDT, (b) WDT, and (c) HPT showed no significant differences between the left and right feet. Mean paired differences were minimal, supporting the use of averaged bilateral values in subsequent analyses.

Bilateral comparisons revealed no significant side-to-side differences for CDT (*p* = 0.707; Wilcoxon signed-rank test), WDT (*p* = 0.638; paired *t*-test), or HPT (*p* = 0.933; Wilcoxon signed-rank test). Mean paired differences were small across all modalities (right minus left: CDT = −0.06 °C, WDT = −0.09 °C, HPT = −0.04 °C), indicating minimal inter-limb variability. These findings support the use of averaged bilateral values for subsequent analyses. Bilateral symmetry of thermal thresholds is illustrated in Fig. 1.

### 3.3 Preliminary reference thermal thresholds

The corresponding central tendency measures and reference ranges are presented in Table 2. Age-stratified descriptive values are provided in Supplementary Table S1. Confidence intervals for the percentile-based limits are also reported.

**Table 2:** Reference values for thermal quantitative sensory testing in healthy Vietnamese adults.

QST parameter	Central tendency	Reference range (2.5–97.5)	95% CI of limits
Cold Detection Threshold (°C)	30.2 (29.5–30.6)	28.01–31.07	Lower: 26.52–28.47; Upper: 30.91–31.59
Warm Detection Threshold (°C)	39.17 ± 1.77	36.05–42.36	Lower: 35.42–36.54; Upper: 41.75–43.01
Heat Pain Threshold (°C)	44.74 (43.65–45.87)	41.12–47.79	Lower: 38.78–42.55; Upper: 46.75–48.02

**Note.** Reference intervals (2.5th–97.5th percentiles) were estimated using non-parametric methods. Confidence intervals for percentile limits were obtained using bootstrap resampling (5000 iterations) and should be interpreted with caution due to the limited sample size.

**Fig. 2:**
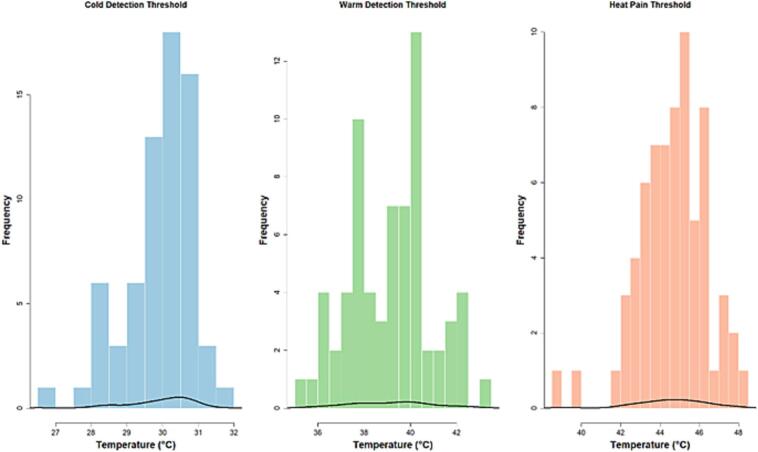
Distribution of thermal sensory thresholds at the foot dorsum.

The distribution of thermal sensory thresholds is shown in Fig. 2. The median CDT was 30.2 °C (IQR: 29.5–30.6), with a reference range of 28.01–31.07 °C. The mean WDT was 39.17 ± 1.77 °C, yielding a reference range of 35.63–42.71 °C. For HPT, the median value was 44.74 °C (IQR: 43.65–45.87), with a reference range of 41.12–47.79 °C. The confidence intervals for these limits were relatively wide, particularly for lower bounds, reflecting uncertainty associated with the sample size.

### 3.4 Correlation and regression analysis

To explore potential demographic influences on thermal QST parameters, we first examined unadjusted associations with age and sex. Visual inspection and correlation analyses demonstrated significant age-related trends for all three parameters, whereas no significant differences were observed between sexes.

**Fig. 3:**
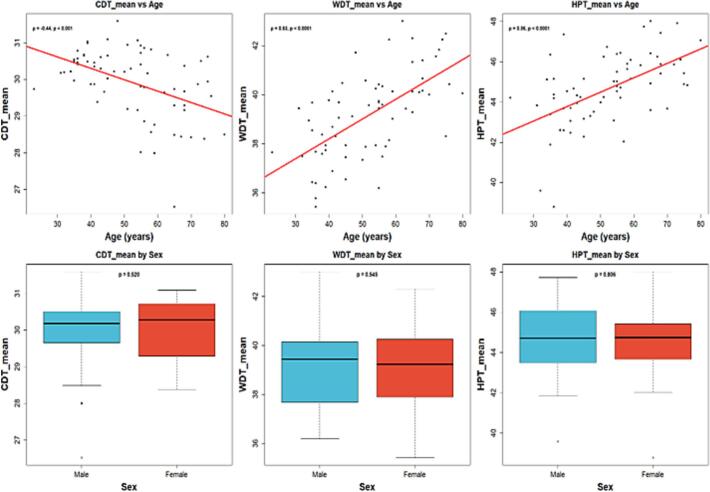
Effects of age and sex on thermal quantitative sensory testing parameters.

Age showed a significant association with all thermal QST parameters (Fig. 3). Increasing age was moderately correlated with CDT (Spearman's ρ = −0.44, *p <* 0.001) and higher WDT (ρ = 0.63, *p <* 0.0001) and HPT (ρ = 0.56, *p <* 0.0001). These findings indicate an age-related decline in thermal sensitivity, with opposite directional effects on cold versus warm and heat pain perception. In contrast, no statistically significant differences were observed between males and females for CDT, WDT, or HPT (all *p* > 0.50). The distributions of thermal thresholds largely overlapped between sexes, suggesting that sex alone did not materially influence thermal sensory thresholds in this healthy Vietnamese cohort.

**Fig. 4:**
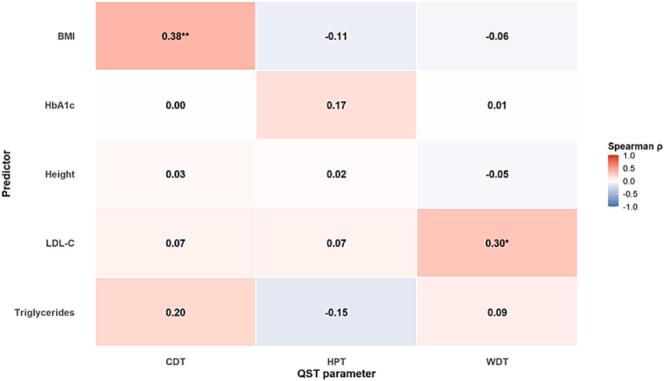
Spearman correlation heatmap between metabolic biomarkers and QST parameters.

Prior to multivariable modeling, univariable correlation analyses were performed to explore the strength and direction of associations between metabolic biomarkers and QST parameters. Spearman correlation coefficients were calculated and visualized using a heatmap. Spearman correlation patterns are illustrated in Fig. 4.

Spearman correlation analysis demonstrated a moderate positive association between BMI and CDT (ρ = 0.38, *p <* 0.01). LDL-C showed a weak but statistically significant positive correlation with WDT (ρ = 0.30, *p <* 0.05).

No significant correlations were observed between HbA1c, height, or triglycerides levels and any of the QST parameters. Overall, most associations were weak in magnitude, suggesting limited univariable relationships between metabolic biomarkers and thermal sensory thresholds.

Based on prior literature, age, sex, height, body mass index (BMI), LDL-C, triglycerides, and HbA1c were considered potential predictors of thermal sensory thresholds. Multivariable linear regression analyses were first performed using a full model including all candidate variables. Subsequently, a reduced model was constructed by retaining variables that were statistically significant or considered clinically relevant, while age and sex were forced into all models as key covariates (see Table 3).

**Table 3:** Multivariable regression model specification (Full model).

Predictor	CDT β (SE)	*p*-value	WDT β (SE)	p-value	HPT β (SE)	p-value
**Intercept**	27.0 (4.08)	<0.001	39.0 (6.59)	<0.001	26.0 (7.41)	<0.001
**Age (years)**	**−0.0337 (0.008)**	**<0.001**	**0.0877 (0.013)**	**<0.001**	**0.0692 (0.015)**	**<0.001**
**Sex (Female vs Male)**	0.385 (0.269)	0.158	−0.679 (0.435)	0.124	0.138 (0.489)	0.779
**Height (m)**	−0.515 (2.05)	0.802	1.32 (3.31)	0.691	5.11 (3.72)	0.175
**BMI (kg/m**^**2**^**)**	0.0866 (0.044)	0.054	−0.0227 (0.071)	0.750	0.0395 (0.080)	0.623
**LDL-C (mg/dL)**	0.00049 (0.0029)	0.864	**0.0121 (0.0046)**	**0.011**	0.00382 (0.0052)	0.465
**Triglycerides (mg/dL)**	0.00154 (0.0013)	0.248	−0.00009 (0.0021)	0.967	−0.00446 (0.0024)	0.068
**HbA1c (%)**	0.565 (0.423)	0.186	−1.33 (0.683)	0.057	1.17 (0.769)	0.132

Model 1:

CDT_mean_ = β_0_ + β_1_(Age) + β_2_(Sex) + β_3_(Height) + β_4_(BMI) + β_5_(LDL-C) + β_6_(Triglycerides) + β_7_(HbA1c) + ϵ.

WDT_mean_ = β_0_ + β_1_(Age) + β_2_(Sex) + β_3_(Height) + β_4_(BMI) + β_5_(LDL-C) + β_6_(Triglycerides) + β_7_(HbA1c) + ϵ.

HPT_mean_ = β_0_ + β_1_(Age) + β_2_(Sex) + β_3_(Height) + β_4_(BMI) + β_5_(LDL-C) + β_6_(Triglycerides) + β_7_(HbA1c) + ϵ.

Model 2:

CDTmean = *β*_*0*_ *+ β*_*1*_*(Age)* *+ β*_*2*_*(Sex) + β*_*3*_*(BMI) + ϵ.*

WDTmean = β_0_+ β_1_(Age) + β_2_(Sex) + β_3_(LDL-C) + ϵ.

HPTmean = β_0_+ β_1_(Age) + β_2_(Sex) + ϵ.

The full multivariable regression results are presented in Table 3 and the reduced model results are summarized in Table 4. In the reduced models, age remained a highly significant predictor across all outcomes, with effect sizes comparable to the full model. Age was negatively associated with CDT (β = −0.0299, *p <* 0.001) and positively associated with WDT (β = 0.0801, *p <* 0.001) and HPT (β = 0.0739, *p <* 0.001), confirming the robustness of age effects. After variable reduction, BMI emerged as an independent predictor of CDT (β = 0.0929, *p* = 0.030), while LDL-C remained independently associated with WDT (β = 0.0122, *p* = 0.007). Sex showed a marginal association with WDT (β = −0.687, *p* = 0.036) but remained non-significant for CDT and HPT. Detailed results are provided in Table 5.

**Table 4:** Reduced multivariable linear regression models for QST parameters.

Predictor	CDT β (SE)	p-value	WDT β (SE)	p-value	HPT β (SE)	p-value
**Intercept**	29.20 (1.11)	**<0.001**	33.80 (0.77)	**<0.001**	41.00 (0.71)	**<0.001**
**Age (years)**	−0.0299 (0.0074)	**<0.001**	0.0801 (0.0119)	**<0.001**	0.0739 (0.0135)	**<0.001**
**Sex (Female vs Male)**	0.392 (0.203)	0.058	−0.687 (0.320)	**0.036**	−0.386 (0.365)	0.295
**BMI**	0.0929 (0.0419)	0.030	–	–	–	–
**LDL-C**	–	–	**0.0122 (0.0043)**	**0.007**	–	–

**Table 5:** Comparison of full and reduced multivariable linear regression models for QST parameters.

Outcome	Model 1 – Full model	Model 2 – Reduced model
Cold Detection Threshold (CDT, °C)	CDT = 26.98–0.0337·Age + 0.385·Female −0.515·Height + 0.0866·BMI + 0.00049·LDL-C + 0.00154·Triglycerides +0.565·HbA1c	CDT = 29.18–0.0299·Age + 0.392·Female +0.0929·BMI
Warm Detection Threshold (WDT, °C)	WDT = 39.01 + 0.0877·Age − 0.679·Female +1.32·Height − 0.0227·BMI + 0.0121·LDL-C − 0.00009·Triglycerides −1.33·HbA1c	WDT = 33.83 + 0.0801·Age − 0.687·Female +0.0122·LDL-C
Heat Pain Threshold (HPT, °C)	HPT = 25.97 + 0.0692·Age + 0.138·Female +5.11·Height + 0.0395·BMI + 0.00382·LDL-C − 0.00446·Triglycerides +1.17·HbA1c	HPT = 40.98 + 0.0739·Age − 0.386·Female

Note:

Female coded as 1 (Female) and 0 (Male).

Age in years; Height in meters; BMI in kg/m^2^; LDL-C and Triglycerides in mg/dL; HbA1c in %.

Model 1 included all prespecified demographic, anthropometric, and metabolic covariates.

Model 2 retained age and sex as a priori covariates and included only predictors showing statistical relevance in the full model.

In the full multivariable models (Model 1), age was a robust predictor of all QST parameters, showing a negative association with CDT (β = −0.0337, *p <* 0.001) and positive associations with both WDT (β = 0.0877, *p <* 0.001) and HPT (β = 0.0692, *p <* 0.001). In contrast, sex, height, and most metabolic biomarkers did not demonstrate consistent independent associations across outcomes, although LDL-C remained significantly associated with WDT (β = 0.0121, *p* = 0.011), and BMI did not reach statistical significance (*p* = 0.054). Multicollinearity was low across predictors (VIF range: 1.16–1.91).

After model reduction (Model 2), the direction, magnitude, and statistical significance of age effects were preserved across all QST parameters, indicating the stability of age as the primary determinant of thermal sensory thresholds. BMI remained independently associated with CDT (β = 0.0929, p = 0.030), while LDL-C remained significantly associated with WDT (β = 0.0122, p = 0.007). Sex did not reach statistical significance for CDT or HPT but showed a modest association with WDT (β = −0.687, p = 0.036).

Overall, the reduced models achieved greater parsimony while retaining the key predictors identified in the full models, supporting their suitability for clinical interpretation and potential application in reference modeling of QST parameters.

## 4 Discussion

### 4.1 The necessity of population-specific normative data

This study provides population-specific reference data for thermal QST parameters at the foot dorsum within a healthy Vietnamese cohort. While the age-dependent trajectory of somatosensory decline observed here mirrors global trends, the absolute thresholds diverge significantly from established Caucasian benchmarks (Rolke et al., 2006) and recent European data using identical Medoc Q-Sense protocols (Cosentino et al., 2023). Specifically, our cohort demonstrates threshold patterns that align more closely with other East Asian populations, highlighting a distinct regional somatosensory profile (Lin et al., 2005; Pan et al., 2024). Such discrepancies underscore the intricate interplay between ethnicity, environmental adaptations, and distinct anthropometric profiles in shaping the somatosensory phenotype (González-Duarte et al., 2016). Consequently, our findings suggest that adopting “off-the-shelf” reference values from Western populations, such as the German DFNS database, could inadvertently introduce systemic diagnostic bias. This practice risks misclassifying healthy Vietnamese individuals, particularly older adults, leading to false-positive diagnoses of small fiber neuropathy (Magerl et al., 2010). These findings support the importance of population-specific reference data for the clinical interpretation of thermal QST parameters (Backonja et al., 2013).

### 4.2 Age-related changes in thermal sensory function

Age was the most consistent factor associated with thermal QST parameters in this study. Increasing age was associated with reduced cold sensitivity and increased warm and heat pain thresholds, reflecting an overall decline in thermal perception. This pattern is consistent with previous studies demonstrating age-related changes in thermal thresholds, particularly at distal sites (Heldestad Lilliesköld and Nordh, 2018; Lin et al., 2005; Zhi et al., 2024). These findings are in line with the concept of length-dependent changes in somatosensory function, with more pronounced effects observed at distal regions such as the foot. Therefore, interpretation of thermal QST parameters should take age-specific reference values into account to reduce the risk of misclassification, particularly in older individuals (Magerl et al., 2010).

### 4.3 The role of sex and anthropometric factors

The influence of sex on distal thermal thresholds appears limited in this context, although further studies with larger samples are needed to confirm this observation. Although some Q-Sense studies have reported sex-related differences in thermal sensitivity (Cosentino et al., 2023), no consistent association between sex and thermal thresholds was observed in this cohort. Even when accounting for confounders, female sex exhibited only a marginal association with lower WDT. These findings are consistent with previous studies suggesting that sex-related differences may be less pronounced at distal sites such as the foot (Magerl et al., 2010; Rolke et al., 2006). In the Vietnamese clinical setting, pooling sex-specific data at the foot may be a pragmatic approach for interpretation, although age- and sex-adjusted models remain preferable in research settings.

### 4.4 Metabolic factors and thermal thresholds

An exploratory finding of this study was that certain metabolic markers, particularly LDL-C, were associated with thermal detection thresholds in this cohort. In the reduced multivariable models, higher BMI was associated with increased CDT, while higher LDL-C was associated with increased WDT. However, these associations were modest and should be interpreted cautiously given the relatively small sample size, the number of predictors included in the models, and the cross-sectional design. Although participants were screened to exclude diabetes and obesity, variability in lipid parameters remained within the cohort. These findings suggest that metabolic factors may contribute to inter-individual variability in thermal thresholds and should be considered when interpreting reference data. Further studies with larger and more metabolically characterized cohorts are needed to clarify these associations.

### 4.5 Bilateral symmetry of thermal thresholds

Our analysis confirms robust bilateral symmetry across all thermal modalities, validating the “internal control” or relative diagnostic approach (Kemler et al., 2000). While absolute population-based thresholds remain the gold standard for detecting systemic polyneuropathies (Backonja et al., 2013), the high degree of inter-limb correlation found here provides a reliable framework for identifying focal or asymmetric small-fiber impairments, where comparison with the contralateral asymptomatic side may provide useful complementary information.

### 4.6 Study limitations

The sample size (*N* = 68) may be underpowered to detect subtle effects of certain variables, such as height or sex. In addition, confidence intervals for percentile-based limits were relatively wide, particularly for lower bounds, reflecting uncertainty associated with the sample size. Therefore, these values should be considered preliminary reference data rather than definitive normative standards. Although participants were recruited from multiple regions, the single-center, convenience sampling design may still limit generalizability. Methodologically, the Method of Limits incorporates reaction time, which may influence threshold estimation, particularly in older participants (Chong and Cros, 2004; Heldestad Lilliesköld and Nordh, 2018). Finally, the cross-sectional nature of this dataset precludes any inferences regarding the longitudinal velocity of sensory decline.

## 5 Conclusions

This study provides preliminary thermal QST reference data at the foot dorsum in a healthy Vietnamese cohort. Age was the most consistent determinant of thermal sensory thresholds, consistent with age-related changes in distal small fiber sensory function. Among metabolic variables, LDL-C demonstrated a consistent association with warm detection thresholds across models, whereas findings for BMI were inconsistent and did not remain significant in the fully adjusted model. These findings suggest that metabolic factors may contribute to variability in thermal thresholds and warrant further investigation in larger studies.

## CRediT authorship contribution statement

**Phan Hoang Phuong Khanh:** Conceptualization, Methodology, Data curation, Formal analysis, Investigation, Writing – original draft. **Pham Dinh San:** Data curation, Investigation, Writing – review & editing. **Phan Hoang Dang Khoa:** Investigation, Writing – review & editing. **Nguyen Huu Cong:** Supervision, Conceptualization, Writing – review & editing. **Nguyen Le Viet Hung:** Methodology, Writing – review & editing. **Nguyen Le Trung Hieu:** Supervision, Project administration, Validation, Writing – review & editing.

## Funding sources

This research did not receive any specific grant from funding agencies in the public, commercial, or non-profit sectors.

## Declaration of competing interest

The authors declare that they have no known competing financial interests or personal relationships that could have appeared to influence the work reported in this paper.

## Appendix A Supplementary Data

Supplementary material: Distribution and normality assessment of thermal QST parameters.
