# Supplementary figures and images for: Foot dorsum thermal quantitative sensory testing thresholds in healthy Vietnamese adults: Reference data
Source: Clin Neurophysiol Pract. 2026 Jun 25;11:562–9. doi: 10.1016/j.cnp.2026.06.009 (PMC13380510; doi:10.1016/j.cnp.2026.06.009)

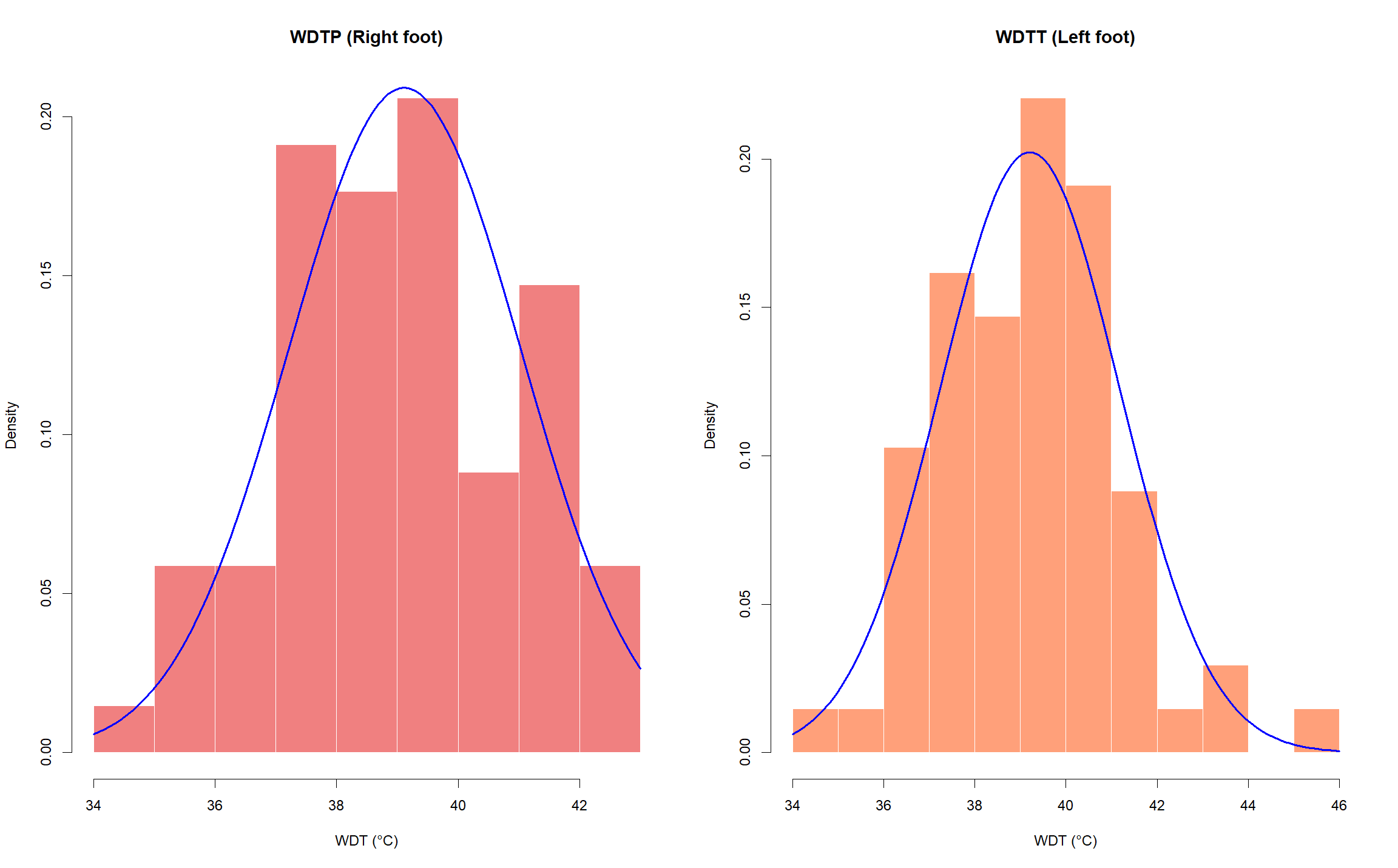


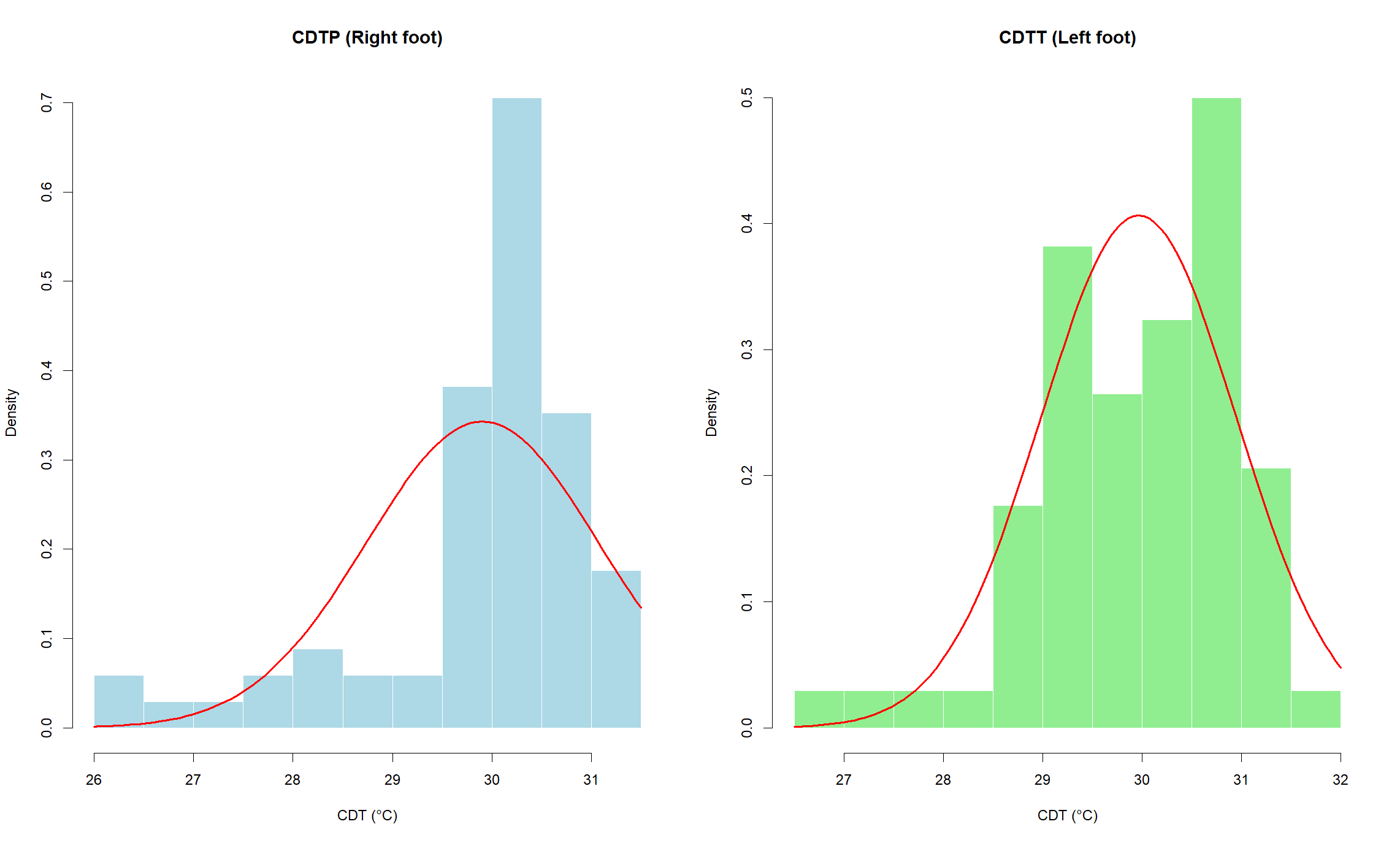


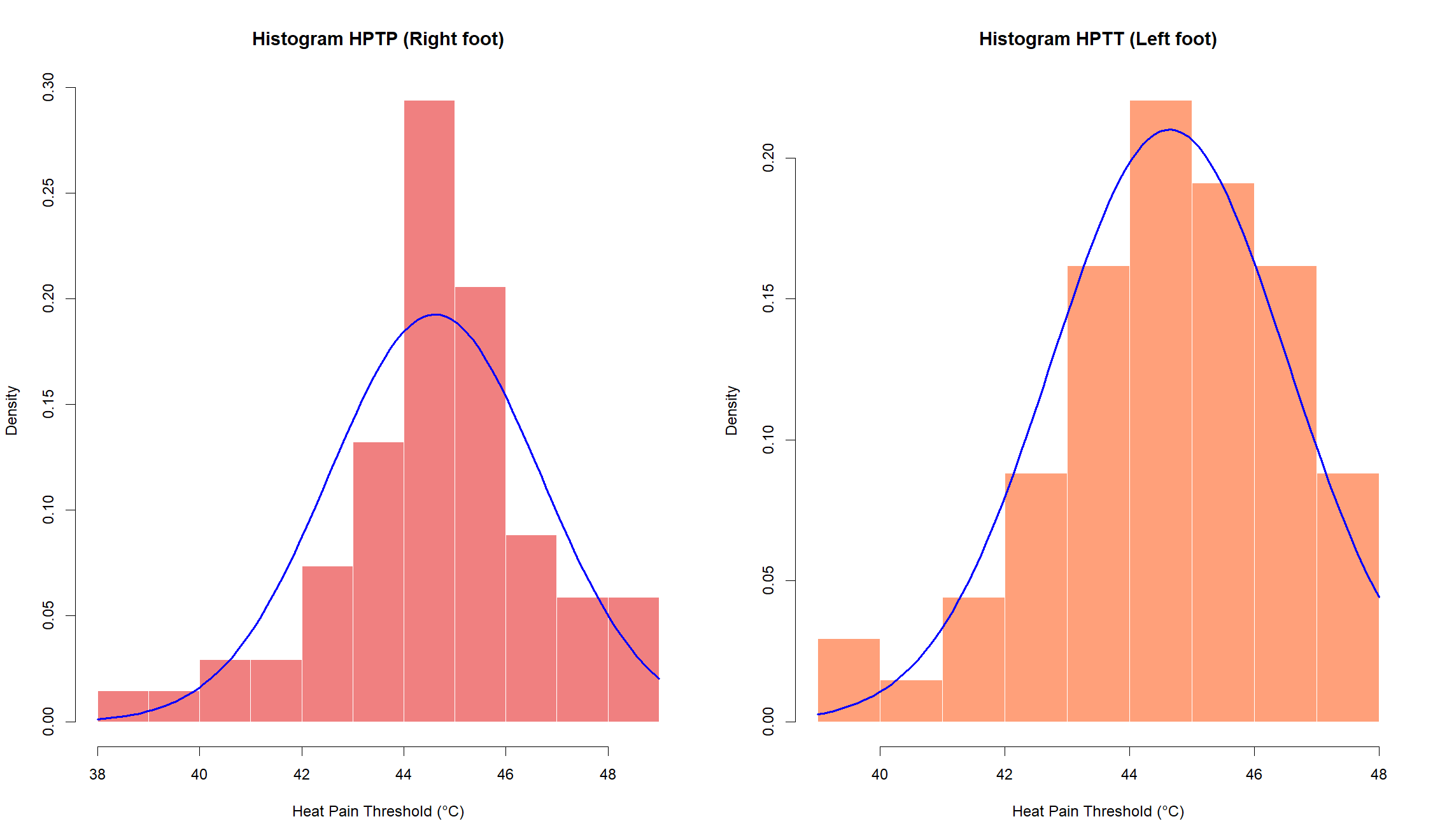


**Supplementary Figure 1. Distribution and normality assessment of thermal QST parameters**

Supplement: Supplementary file 1 — Supplementary material: Distribution and normality assessment of thermal QST parameters. [file mmc1.docx]
