# Supplementary material for: Foot dorsum thermal quantitative sensory testing thresholds in healthy Vietnamese adults: Reference data
Source: Clin Neurophysiol Pract. 2026 Jun 25;11:562–9. doi: 10.1016/j.cnp.2026.06.009 (PMC13380510; doi:10.1016/j.cnp.2026.06.009)
Supplement: Supplementary file 2 — Supplementary Table S1 [file mmc2.docx]

## Supplementary Table S1

**Age-stratified descriptive values of thermal quantitative sensory testing parameters in healthy Vietnamese adults**

| Age group (years) | n | CDT | WDT | HPT |
| --- | --- | --- | --- | --- |
| 18–39 | 17 | 30.5 ± 0.34 | 37.6 ± 1.22 | 43.4 ± 2.14 |
| 40–59 | 32 | 30.0 ± 0.90 | 39.0 ± 1.45 | 44.5 ± 1.25 |
| ≥60 | 19 | 29.3 ± 1.02 | 40.8 ± 1.24 | 45.9 ± 1.29 |

**Note.** Values are presented for descriptive purposes only and should not be interpreted as age-specific reference intervals. If the original data confirm non-normal distributions, CDT and HPT should preferably be reported as median (IQR), while WDT should be reported as mean ± SD.
